# Supplementary material for: mRNA condensation fluidizes the cytoplasm
Source: bioRxiv. 2023 Jul 15:2023.05.30.542963. Originally published 2023 May 31. Preprint. [Version 2] doi: 10.1101/2023.05.30.542963 (PMC10312499; doi:10.1101/2023.05.30.542963)
Supplement: 1 [file NIHPP2023.05.30.542963V2-supplement-1.pdf]

bioRxiv preprint doi: https://doi.org/10.1101/2023.05.30.542963; this version posted July 15, 2023. The copyright holder for this preprint (which was not certified by peer review) is the author/funder, who has granted bioRxiv a license to display the preprint in perpetuity. It is made available under aCC-BY 4.0 International license.

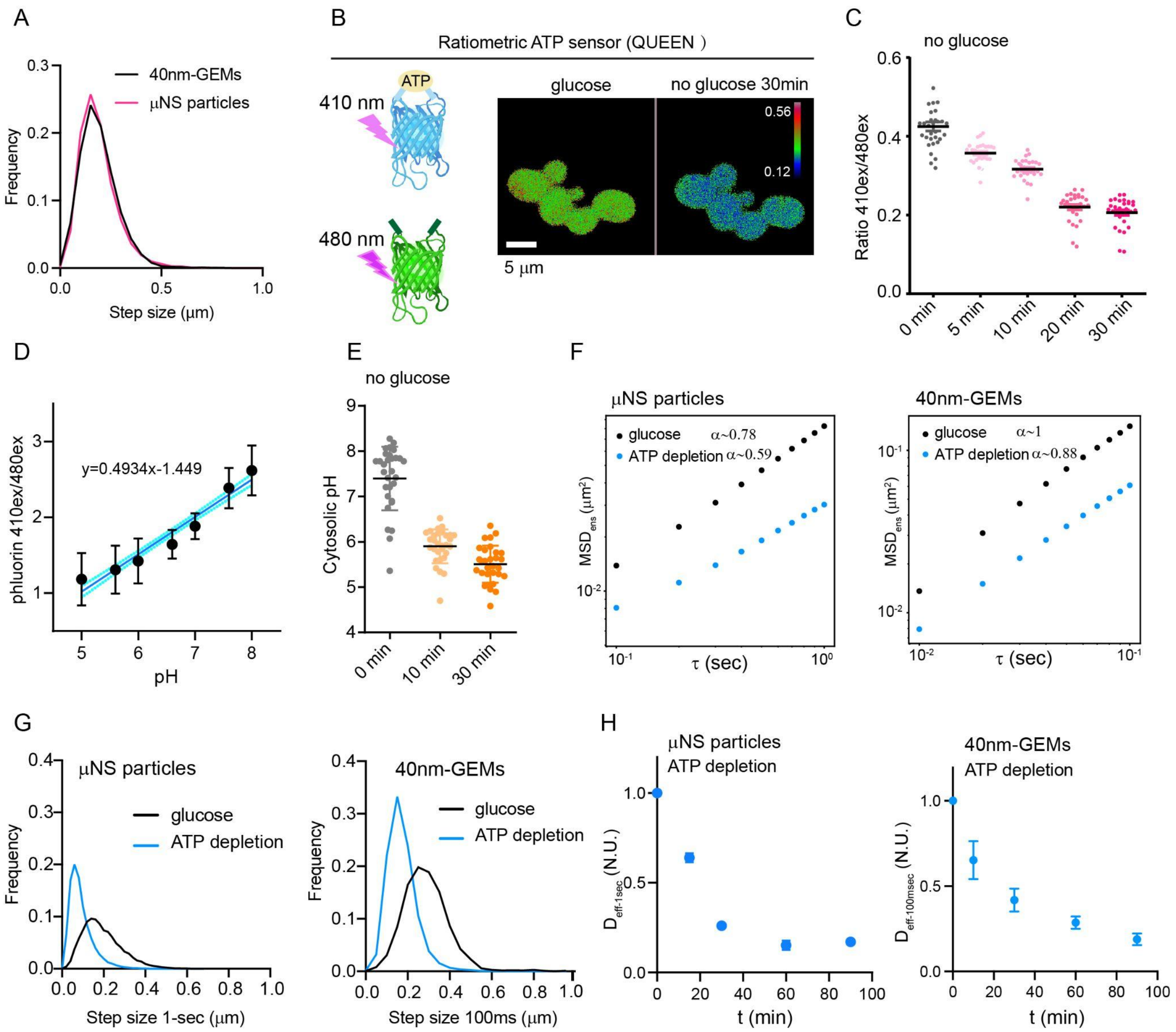

Supplementary figure 1. ATP reduction and cytoplasmic acidification decrease mesoscale macromolecular diffusivity, related to Figure 1. A. Step size distribution of 40nm-GEMs in 100 ms and µNS particles in 1 second. **B.** Graphic illustration of QUEEN sensor. ATP QUEEN sensor displays a bimodal excitation spectrum with peaks at 410 nm (ATP-bound) and 480 nm (ATP-unbound) and an emission maximum at 510 nm. Representative QUEEN ratio (410 nm ex/480 nm ex) images of yeast cells in glucose rich and glucose depleted conditions. The QUEEN ratio is pseudo-colored. **C.** Time course analysis of the QUEEN ratio after glucose depletion (n=30 cells, mean±SEM). **D.** pH calibration curve determined using permeabilized *S. cerevisiae* cells expressing cytosolic pHluorin sensor. A linear model was fitted to determine the

standard curve (n=30 cells for each pH condition). **E.** The intracellular pH was quantified based on the standard curve of pHluorin sensor in *S. cerevisiae* cells upon acute glucose starvation (n=30 cells, mean±SEM). **F.** Ensemble-averaged mean-squared displacement (MSD) versus time delay (τ), log10 scale. A linear model was fitted to determine the anomalous exponent α values for glucose rich (glucose) or ATP depleted (ATP depletion) for μNS particles and 40nm-GEMs at 30 minutes (ATP depleted condition: synthetic complete medium buffer at pH 5.5 without glucose, 20 mM 2-deoxyglucose and 10 μM antimycin A and 80 mM sorbitol were supplemented. Trajectories analyzed for μNS particles, glucose: n=16373 trajectories; ATP depletion: n=16373 trajectories; For 40nm-GEMs, glucose: n=2620 trajectories; ATP depletion: n=3176 trajectories). **G.** Step size distribution of μNS particles in one second time scale, 40nm-GEMs in 100 msec time scale in glucose rich and ATP depleted conditions at 30 minutes (For μNS particles, glucose: n=16373 trajectories; ATP depletion: n=16373 trajectories; For 40nm-GEMs, glucose: n=2620 trajectories; ATP depletion: n=3176 trajectories). **H.** Fold change of median effective diffusion coefficients at one sec time scale for μNS particles, and 100 msec ($D_{eff\text{-}100ms}$) for 40nm-GEMs from 3 biological replicate experiments (mean ± SEM.) upon ATP depletion conditions in *S. cerevisiae* cells.

bioRxiv preprint doi: https://doi.org/10.1101/2023.05.30.542963; this version posted July 15, 2023. The copyright holder for this preprint (which was not certified by peer review) is the author/funder, who has granted bioRxiv a license to display the preprint in perpetuity. It is made available under aCC-BY 4.0 International license.

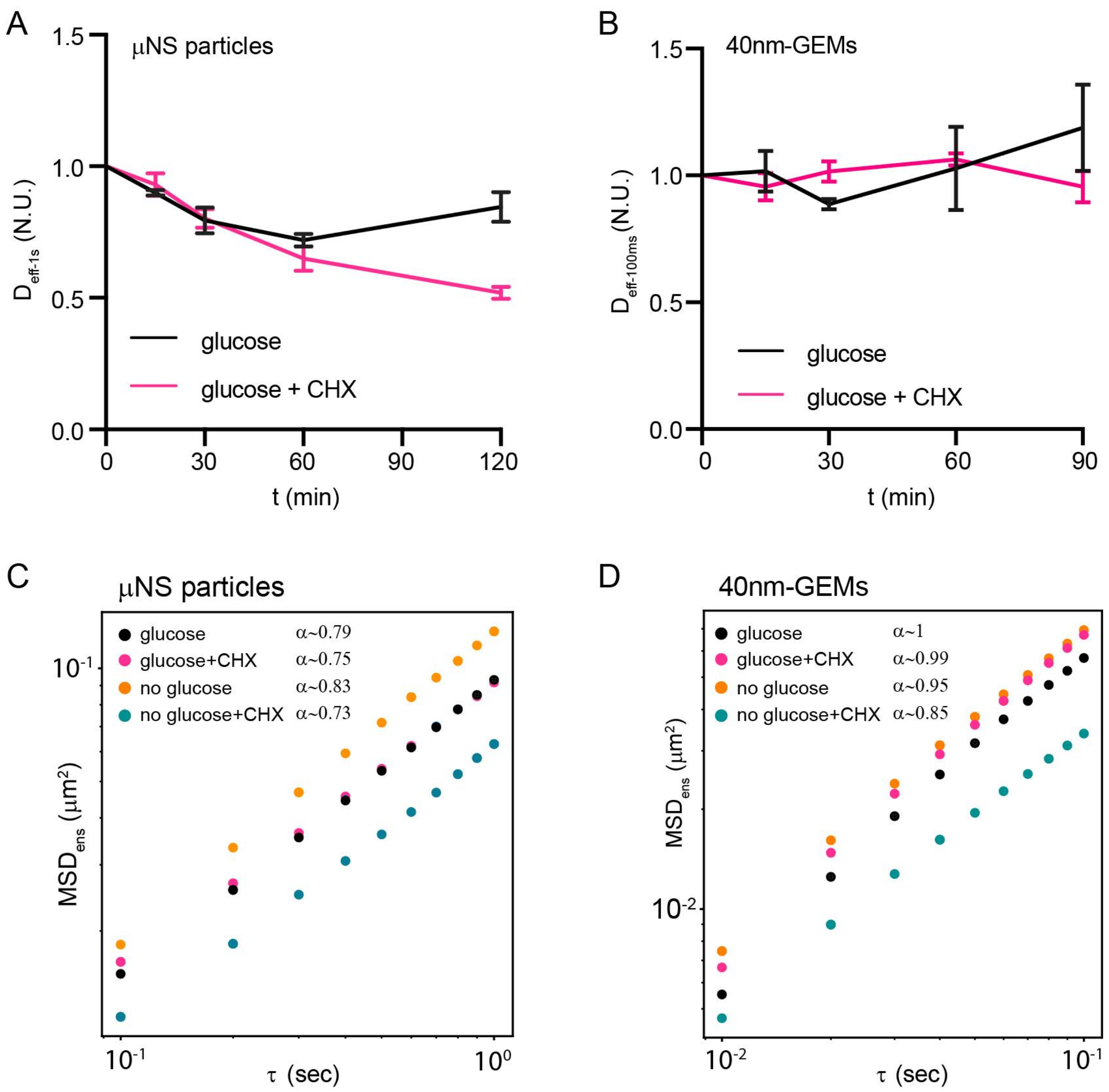

Supplementary figure 2. Mesoscale μNS particles and 40nm-GEMs dynamics in wild type *S. cerevisiae* cells, related to figure 2. A. Fold change of median effective diffusion coefficients for μNS particles from 3 biological replicate experiments in the indicated condition (CHX: cycloheximide, mean ± SEM.). **B.** Fold change of median effective diffusion coefficients for 40nm-GEMs from 3 biological replicate experiments in the indicated conditions (CHX: cycloheximide, mean ± SEM.). **C.** Ensemble-averaged mean-squared displacement (MSD) versus time delay (τ), log10 scale. A linear model was fit to determine the anomalous exponent α values for μNS particles in the indicated conditions at 30 minutes (n>10000 trajectories in each condition, CHX: cycloheximide). **D.** Ensemble-averaged mean-squared displacement (MSD) versus time delay (τ), log10 scale. A linear model was fit to determine the anomalous

exponent α values for 40nm-GEMs in the indicated condition at 30 minutes (n>5000 trajectories in each condition, CHX: cycloheximide).

bioRxiv preprint doi: https://doi.org/10.1101/2023.05.30.542963; this version posted July 15, 2023. The copyright holder for this preprint (which was not certified by peer review) is the author/funder, who has granted bioRxiv a license to display the preprint in perpetuity. It is made available under aCC-BY 4.0 International license.

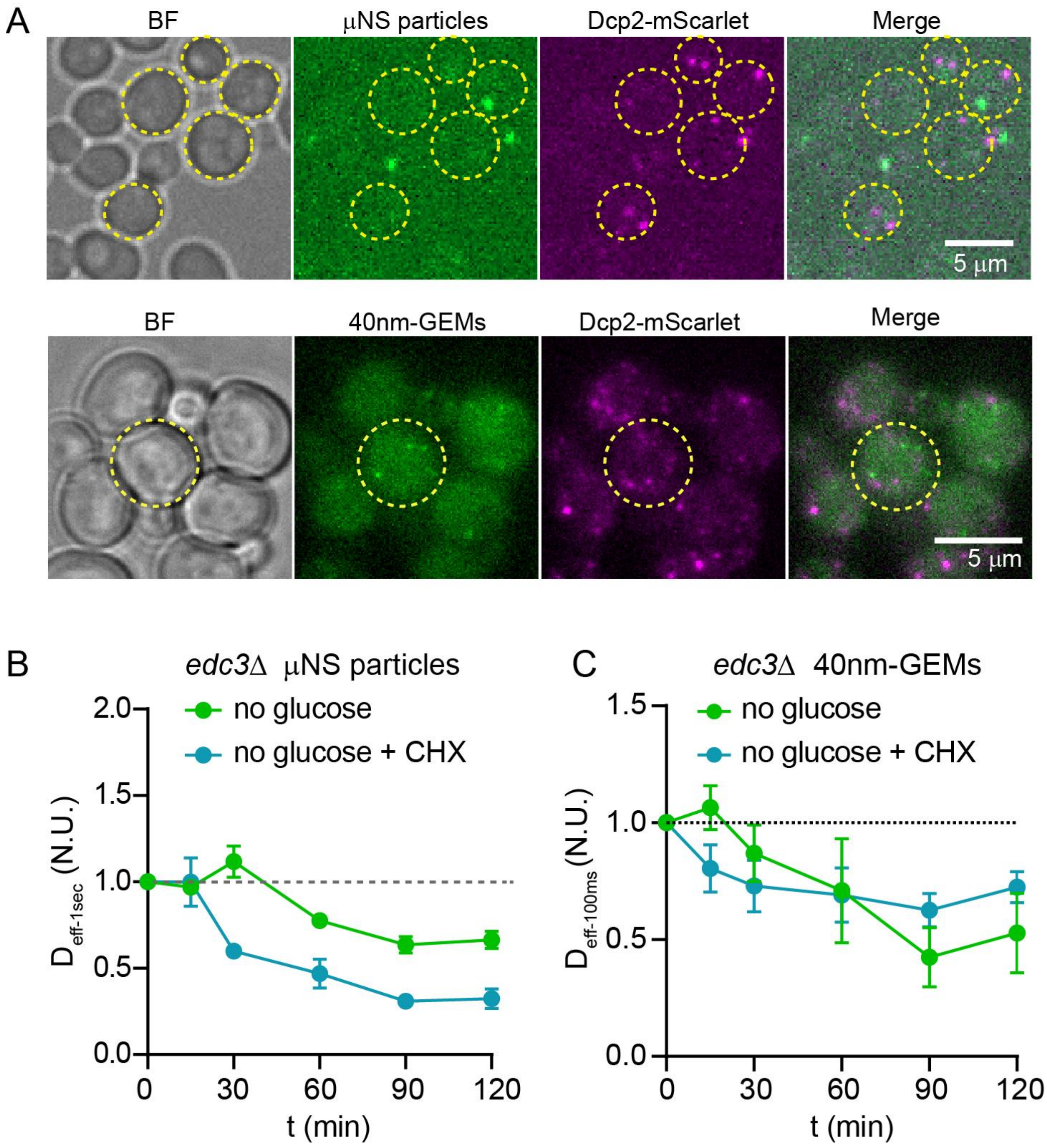

Supplementary figure 3. Mesoscale µNS particles and 40nm-GEMs behavior in *edc3Δ S. cerevisiae* cells, related to figure 3. A. P-bodies (Dcp2-mScarlet marker) are not colocalized with µNS particles or 40nm-GEMs (BF = Bright field). **B.** Fold change of median effective diffusion coefficients for µNS particles from 3 biological replicate experiments at the indicated conditions (CHX: cycloheximide, mean ± SEM.). **C.** Fold change of median effective diffusion coefficients for 40nm-GEMs from 3 biological replicate experiments at the indicated conditions (CHX: cycloheximide, mean ± SEM.).

bioRxiv preprint doi: https://doi.org/10.1101/2023.05.30.542963; this version posted July 15, 2023. The copyright holder for this preprint (which was not certified by peer review) is the author/funder, who has granted bioRxiv a license to display the preprint in perpetuity. It is made available under aCC-BY 4.0 International license.

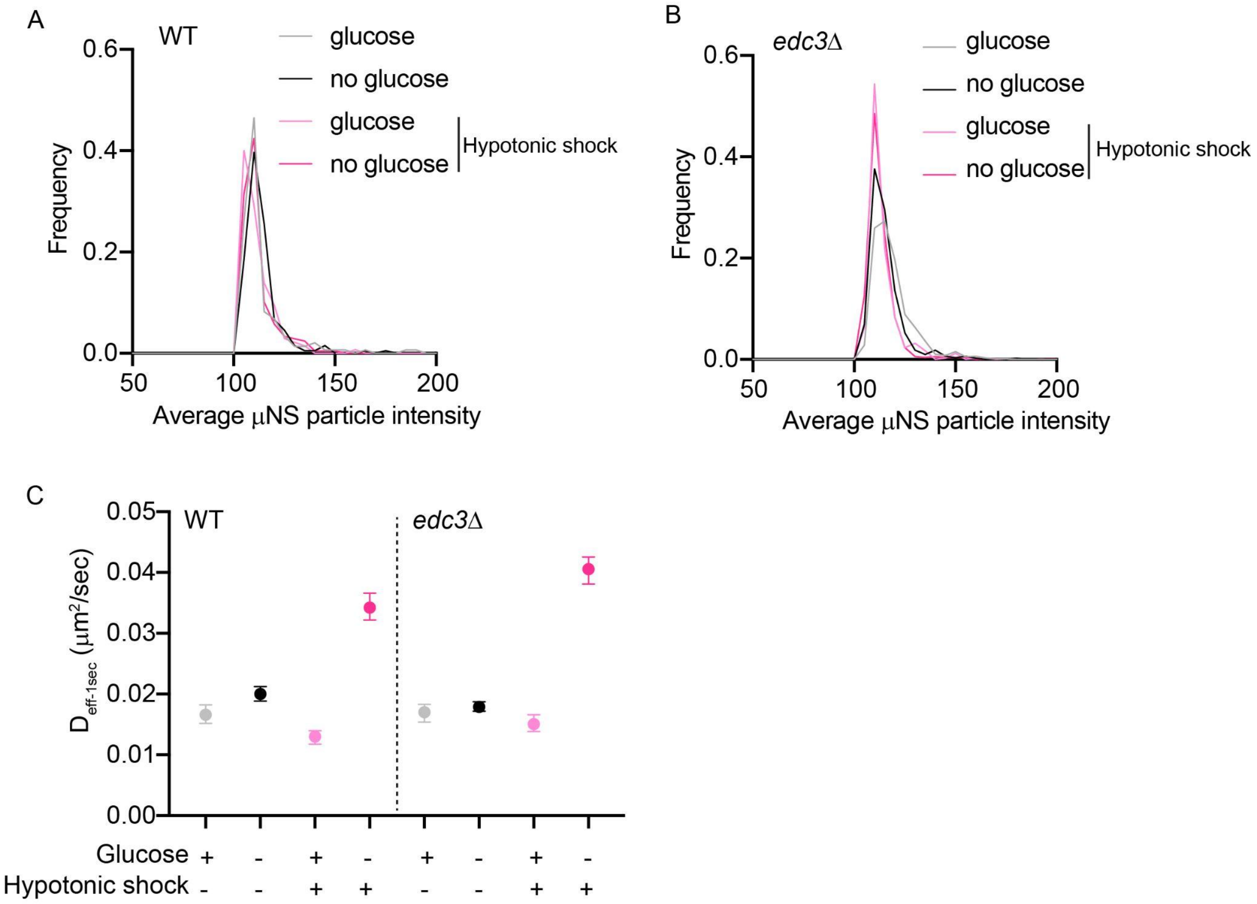

Supplementary figure 4. Mesoscale µNS particles diffusivity increases upon hypotonic shock, related to figure 4. A. Distribution of average particle intensities for µNS particles in the indicated conditions (n>700 trajectories in each condition) for wildtype and **B.** *edc3Δ* cells. **C.** Effective diffusion coefficients at 1-second time scale for µNS particles in the indicated conditions in wildtype and *edc3Δ* cells. All glucose starvation results were assessed 5 min after starvation (n>700 trajectories in each condition, median ± 95% confidence interval).

bioRxiv preprint doi: https://doi.org/10.1101/2023.05.30.542963; this version posted July 15, 2023. The copyright holder for this preprint (which was not certified by peer review) is the author/funder, who has granted bioRxiv a license to display the preprint in perpetuity. It is made available under aCC-BY 4.0 International license.

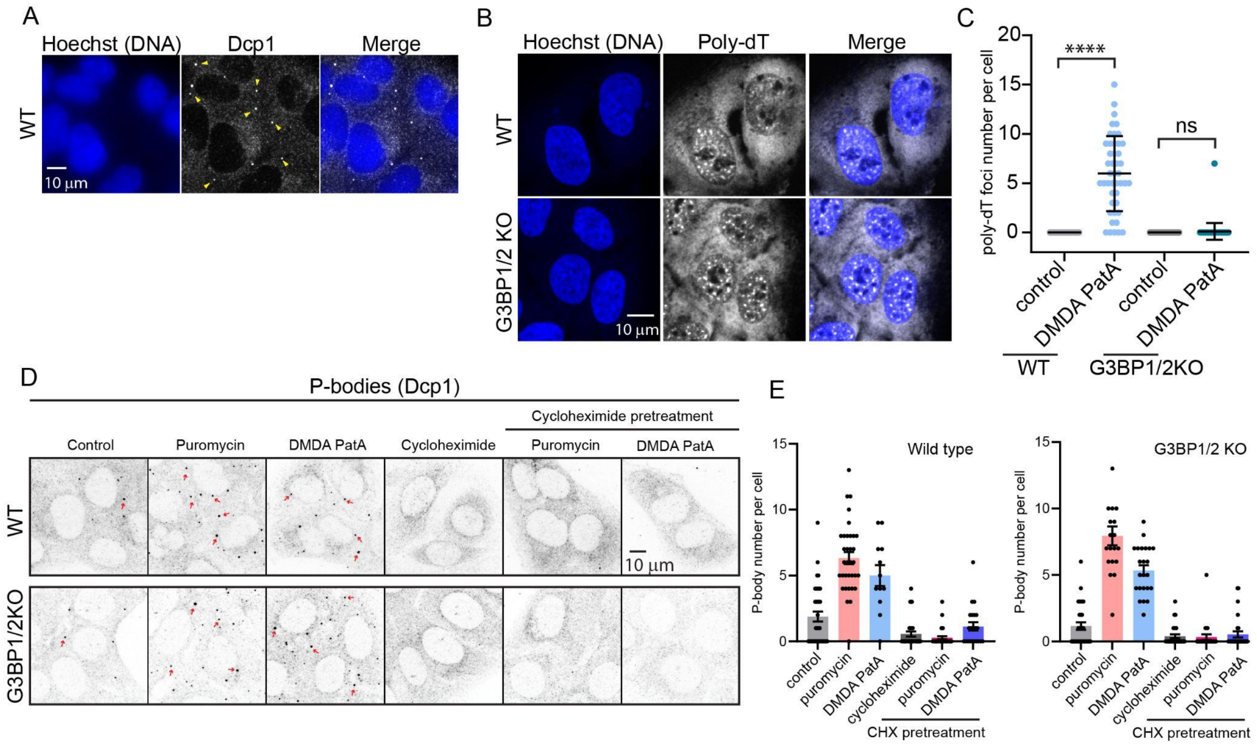

Supplementary figure 5. Characterization of P-bodies and mRNA localization upon treatment with translation inhibitors, related to figure 5. A. Representative

immunofluorescence images of P-bodies (Dcp1) in U2OS wild type cells in the absence of stress. Hoechst dye was used to stain DNA. **B.** Representative poly-dT FISH images for poly(A) RNAs in wild type and G3BP1/2 KO U2OS cells without any drug treatment. **C.** Quantification of poly-dT FISH foci number in the cytoplasm with the indicated conditions (WT control: n=57 cells; WT DMDA-PatA n=45 cells; G3BP1/2 KO control n=76 cells; G3BP1/2 KO DMDA-PatA n=67 cells, mean ± SD. Statistical comparison was by a one-way ANOVA test. ns=not significant, *p<0.05, **p<0.01, ****p<0.0001). **D.** Representative immunofluorescence images of P-bodies (Dcp1) in U2OS wild type and G3BP1/2KO cells. **E.** Quantification of Dcp1 foci (P-bodies) number in U2OS wild type and G3BP1/2KO cells with the indicated translation inhibitors (WT control n=35; WT puromycin n=34; WT DMDA-PatA n=12; WT cycloheximide n=32; WT Cycloheximide pretreatment puromycin n=35; WT Cycloheximide pretreatment DMDA-PatA n=22. G3BP1/2 KO control n=31; G3BP1/2 KO puromycin n=19; G3BP1/2 KO DMDA-PatA n=23; G3BP1/2 KO cycloheximide n=28; G3BP1/2 KO Cycloheximide pretreatment puromycin n=24; G3BP1/2 KO Cycloheximide pretreatment DMDA-PatA n=28. mean ± SD. Statistical comparison was by a one-way ANOVA test. ns=not significant, *p<0.05, **p<0.01, ****p<0.0001).
